# Wearable Robotic Gait Training in Persons with Multiple Sclerosis: A Satisfaction Study

**DOI:** 10.3390/s21144940

**Published:** 2021-07-20

**Authors:** Diego Fernández-Vázquez, Roberto Cano-de-la-Cuerda, María Dolores Gor-García-Fogeda, Francisco Molina-Rueda

**Affiliations:** 1 International Doctorate School, Rey Juan Carlos University, 28922 Madrid, Spain; diego.fernandez@urjc.es; 2 Department of Physical Therapy, Occupational Therapy, Physical Medicine and Rehabilitation, Faculty of Health Sciences, Rey Juan Carlos University, Alcorcón, 28922 Madrid, Spain; francisco.molina@urjc.es; 3 Madrid Foundation against Multiple Sclerosis, 28029 Madrid, Spain; lola.gor84@gmail.com

**Keywords:** exoskeletons, gait, multiple sclerosis, physical therapy, rehabilitation, satisfaction, technology, robot

## Abstract

Wearable exoskeletons have showed improvements in levels of disability and quality of life in people with neurological disorders. However, it is important to understand users’ perspectives. The aim of this study was to explore the patients’ and physiotherapists’ satisfaction from gait training with the EKSO GT^®^ exoskeleton in people with multiple sclerosis (MS). A cross-sectional study with 54 participants was conducted. Clinical data and self-administered scales data were registered from all patients who performed sessions with EKSO GT^®^. To evaluate patients’ satisfaction the Quebec User Evaluation with Assistive Technology and Client Satisfaction Questionnaire were used. A high level of satisfaction was reported for patients and for physiotherapists. A moderate correlation was found between the number of sessions and the patients’ satisfaction score (rho = 0.532; *p* < 0.001), and an excellent correlation between the physiotherapists’ time of experience in neurology rehabilitation and the satisfaction with the possibility of combining the device with other gait trainings approaches (rho = 0.723; *p* = 0.003). This study demonstrates a good degree of satisfaction for people with MS (31.3 ± 5.70 out of 40) and physiotherapists (38.50 ± 3.67 out of 45 points) with the EKSO GT^®^. Effectiveness, safety and impact on the patients’ gait were the most highly rated characteristics of EKSO GT^®^. Features such as comfort or weight of the device should be improved from the patients’ perspectives.

## 1. Introduction

Multiple sclerosis (MS) is a chronic autoimmune disease of the central nervous system in which inflammation, demyelination, and axonal loss occurs even in early stages of the disease. MS is the main cause of disability in young adults with a prevalence of 140 and 108 per 100,000 individuals in North America and Europe respectively [1]. Walking disability is one of the most common symptoms associated to MS, and one of the most challenging from the perspective of the patients and their caregivers. Between the 50–80% of the patients have balance and gait dysfunctions and over 50% fall at least once a year, and also 50% of the patients need walking aid requirements after 15 years [2].

Neurorehabilitation can help improve gait disturbances using different approaches, such as robot-assisted therapy which has been recently developed and is now being implemented in rehabilitation services [3]. The use of exoskeletons provides some benefits compared to conventional therapy, such as the possibility to reproduce physiological gait patters and to allow for more intensive therapy with more number of repetitions, maximizing time and decreasing the effort for both patient and therapist. In addition, exoskeletons can assess gait parameters during therapy [4].

There are two kinds of exoskeletons: grounded exoskeletons, which allow to walk on a treadmill, or over-ground wearable exoskeletons [3]. Regarding MS, there are only a few studies that analyse the effects of gait training using exoskeletons, and most of them have been carried out with fixed exoskeletons. These studies have shown that robot-assisted therapy can improve spatiotemporal gait parameters, level of disability, quality of life and balance [5,6]. Grounded exoskeletons have certain disadvantages compared to wearable exoskeletons: the patient walks in place and does not move over ground, they do not allow turning or movement upstairs, and they hinder the opportunity to socialize easily with the environment [3,7,8]. Concerning wearable exoskeletons, evidence is still very scarce, although some improvements have been proved regarding gait velocity, fatigue, and quality of life in some studies with small sample sizes [9,10,11,12].

EKSO GT^®^ is an over-ground wearable exoskeleton developed and commercialized to train and improve gait in people with neurological diseases [13]. This device reproduces physiological gait minimizing compensatory patterns and guiding recovery. It is indicated for patients who have gait disturbances caused by neurological conditions, in both people who already ambulate, and those who are not able to walk, and it must always be guided by a certified therapist [14]. As described, most studies on robotic exoskeletons to date have focused on biomechanics, safety, and clinical outcomes. Related to MS, there is evidence that therapy with EKSO GT^®^ is associated with large improvements in functional mobility and cognitive processing compared to a conventional overground walking [15,16]. Furthermore, only a few studies have examined the perspectives of patients, and even fewer have investigated physiotherapists’ perspectives about exoskeletons in people with MS. 

To our knowledge, no published study has specifically investigated the usability of the EKSO GT^®^ exoskeleton for MS. Thus, the main objective of this study was to explore the patients’ and physiotherapists’ satisfaction from gait training with the EKSO GT^®^ exoskeleton in people with MS.

## 2. Materials and Methods

### 2.1. Design

A cross-sectional study was conducted at the “Madrid Foundation against Multiple Sclerosis” (Madrid, Spain). Strengthening the Reporting of Observational Studies in Epidemiology (STROBE) [17] guidelines were followed to standardize the reporting of this research. Informed consent was obtained from the patients prior to their inclusion in the study, which was conducted in accordance with the Declaration of Helsinki. A Local Ethical Committee approved the study (04/20). 

### 2.2. Participants

Subjects were retrospectively screened for eligibility from June to December 2020 at the “Madrid Foundation against Multiple Sclerosis” for patients who met the requirements for this study.

The inclusion criteria were as follows: (1) age over 18 years old, (2) one-year diagnosis of MS that was made according to the McDonald criteria [18], (3) 24 points or more on the MMSE [19], and (4) had performed at least 3 sessions of 45 min with the EKSO GT^®^. The exclusion criteria were: (1) diagnosis of neurological injury other that MS, (2) exacerbations or treatment with corticosteroids or botulinum toxin in the previous three months, (3) visual or cognitive disturbances that hinder gait training, (4) severe concurrent medical disease, illness, or condition that interfere with ability to participate, or (5) present any contraindication from the manufacturer of the EKSO GT^®^ (ulcers, unstable spine, colostomy, severe spasticity—equal or more than 4 points in the modified Ashworth scale, or pregnancy).

The physiotherapists that participated in the study met the following criteria: (1) to carry out neurorehabilitation therapies at the “Madrid Foundation against Multiple Sclerosis”. (2) to perform rehabilitation sessions with patients who received therapy with EKSO GT^®^, and (3) to supervise a completed session with EKSO GT^®^ with each patient.

### 2.3. Sample Size

The usability sample size was calculated with G*Power software (version 3.1.9.6). We retrospectively established the following parameters to obtain the sample size using a correlation model: two tails, an expected correlation ρ H1 of 0.7, an error alpha of 0.05, a power of 0.90, and a minimally acceptable ρ H0 of 0.3, resulting in a sample size requirement of 36 participants.

### 2.4. Outcomes Measures

The following information was collected for all patients: age, gender, weight, height, type of MS, years from the diagnosis, Expanded Disability Status Scale (EDSS) and type of support device.

Subsequently, the subjects completed these questionnaires: *Quebec User Evaluation with Assistive Technology (QUEST 2.0)* [20] and *Client Satisfaction Questionnaire (CSQ-8)* [21] assess their degree of satisfaction with the EKSO GT^®^. The following self-administered scales were used: *Multiple Sclerosis Walking Scale-12 (MSWS-12)* [22], *Modified Fatigue Impact Scale (MFIS)* [23], *Multiple Sclerosis Impact Scale (MSIS-29)* [24], *Hospital Anxiety and Depression Scale (HADS)* [25] and *36-Item Shor Form Health Survey questionnaire (SF36)* [26].

*QUEST 2.0* is a standardized form comprising 12 items that identifies the user’s satisfaction and dissatisfaction in relation to assistive technology and service. Eight items are related to user satisfaction with the assistive devices, and the remaining four items, which assess service delivery, were omitted considering that they could not be adequately assessed. *QUEST 2.0* uses a five-level response scale from 1 (not satisfied at all) to 5 (very satisfied). QUEST has been demonstrated to be a valid and reliable assessment tool in people with MS [27]. 

The *CSQ-8* is a self-report questionnaire that assesses the overall level of satisfaction with the service received. It is comprised of eight items that is scored on a scale, ranging from 1 to 4, where a higher score indicates greater satisfaction. The *CSQ-8* has a robust reliability and validity to measure the satisfaction of an intervention [28].

The *MSWS-12* is a commonly adopted patient-reported outcome measure used to assess the extent to which MS impacts the walking ability. It has been proved to be a reliable, valid, and sensitive measure [29].

The *MFIS* is a self-report questionnaire to assess the effects of fatigue on quality of life in terms of physical, cognitive, and social functioning. The MFIS was found to be highly reliable and valid in people with MS [30].

The *MSIS-29* is a patient-reported outcome that attempts to assess both physical and psychological impact of MS on quality of life. The psychometric properties of this scale were studied, giving an adequate result in terms of reliability and validity [31].

The *HADS* is a brief self-reporting two-dimensional questionnaire developed to screen for levels of anxiety and depression of patients. The psychometric properties of *HADS* are good, so it is a useful instrument to be included in the study with people with MS [32].

The *SF36* is an outcome instrument widely used to assess the health-related quality of life It is a multidimensional generic instrument validated for several pathologies including MS. The physical component summary of the SF36 evaluates functional capacity, physical aspects, pain, and general health, whereas its mental component summary assesses vitality, social functioning, emotional aspects, and mental health [33]. 

The physiotherapists who participated in the study were asked to fill out a 9-item questionnaire to evaluate their satisfaction (Table 1). Each item of the questionnaire was rated on a five-point Likert scale from 1 (not satisfied at all) to 5 (very satisfied). Information related to age, gender, and years of experience in neurologic rehabilitation and with people with MS was also recorded.

### 2.5. Main Description of the Exoskeleton

EKSO GT^®^ is a lower limb wearable exoskeleton designed for gait rehabilitation. EKSO GT^®^ is equipped with 4 electric motors to move the four degrees of freedom, the flexion and extension of the hip and knee. Gait parameters such as step length, step height or the swing time of the leg can be modified. All robotic movements and parameters are managed using an external controller stored on a magnetic seat at the backpack.

### 2.6. Procedure of the Sessions 

In the first session, an initial assessment was carried out to verify that the patients meet the inclusion criteria of the device. Muscular strength, spasticity, joint angles of the upper and lower limbs, and the anthropometric measurements necessary to fit the exoskeleton were recorded.

In the second session, the first standing and walking was performed. Before walking, all the patients performed the body weight shifting activity from the EKSO GT^®^ Pregait program. The default settings during this first session were the “FirstStep” mode, a completed and bilateral assistance supported by EKSO GT^®^ and a rolling walker.

In the third session, the walking rehabilitation training was based on the patients’ needs progression according to the recommendations the EKSO GT^®^ clinical training guide. Adverse effects, step number, standing time, walking time, the amount of power contribution to one or both legs during walking were recorded. 

A minimum of 100 steps was required to consider the initiation sessions completed. From the third session, and in agreement with the rehabilitation team, EKSO GT^®^ was introduced as a regular therapy once a week or every 2 weeks. The total number of sessions performed by each participant was recorded. The training protocol was administered by a physiotherapist who had completed the device manufacturer’s training program.

### 2.7. Statistical Analysis 

Statistical analysis was performed using the SPSS statistical software (version 27.0). 

Descriptive statistics were used to analyze quantitative data (mean ± standard deviation) and qualitative data (frequency and proportions). A correlation analysis between dependent variables (QUEST 2.0, CSQ8, physiotherapists’ questionnaire) and independent variables (the rest of scales and social-demographic data) was performed to assess the association between them. Shapiro–Wilk and Kolmogórov–Smirnov test were used to examine normality of clinical scores. Spearman correlation coefficient was used (with 95% Confident Intervals) due to the samples violated the statistical normality. Correlation coefficients of 0.00–0.30 were interpreted as poor, those of 0.30 to 0.70 as moderate, and those of 0.70 or higher as excellent [34]. Significance level was set at 0.05.

## 3. Results

**Figure 1:**
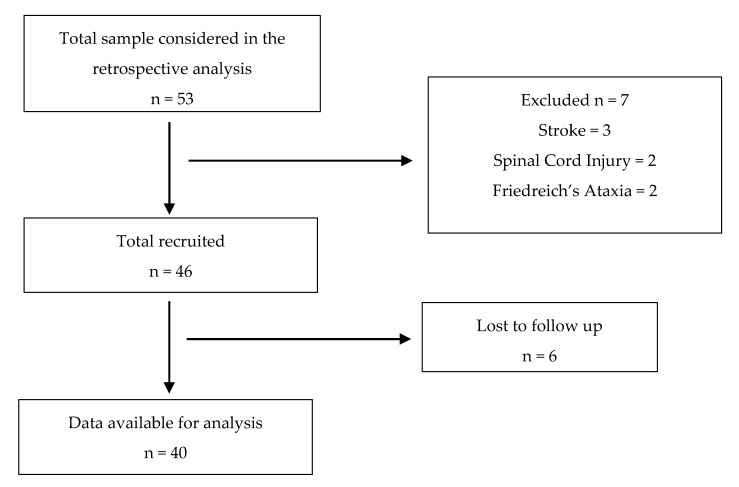
Strobe flowchart.

Fifty-three patients with MS were initially included in the present study, but 40 patients completed all sessions (21 women and 19 men, 49.5 ± 7.99 years, 170.43 ± 8.8 cm, 68.83 ± 14.85 kg, EDSS of 6.38 ± 1.5, sessions performed of 13.53 ± 9) (Figure 1). Regarding the type of EM, 9 patients presented relapsing–remitting, 20 secondary–progressive and 11 primary–progressive.

**Figure 2:**
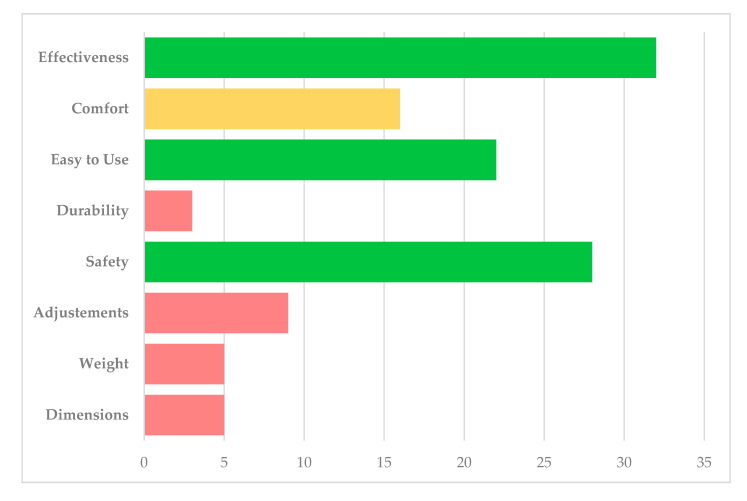
Best rated items by patients on the Quebec User Evaluation of Satisfaction with assistive Technology (QUEST) test. The percentage of each topic refers to the number of participants who considered that characteristic as relevant (red < 10, yellow 10–20, green > 20).

The average total score of the QUEST 2.0 was 31.3 ± 5.70 out of 40, and the CSQ-8 obtained 26.28 ± 4.68 out of 32 points. The mean scores of all scales are included in Table 2. The parameter selected the most was the effectiveness, followed by safety. Figure 2 shows the best-rated items selected by the patients.

Fourteen physiotherapists participated in the present study, 12 women and 2 men, with a mean age of 28.43 ± 4.55 years, an average time of experience in neurology rehabilitation of 5.54 ± 4.54 years, and an average time of experience in rehabilitation in people with MS of 3.29 ± 1.76 years. 

The mean score of the physiotherapists’ satisfaction questionnaire was 38.50 ± 3.67 points out of 45 points. Scores for each item are showed in Table 3.

The correlation analysis for MS participants showed poor associations between clinical variables, except for the association between the CSQ-8 and the number of sessions performed, which had a good coefficient (rho = 0.532; *p* < 0.001). There is also, a moderate correlation between MSIS and the items comfort and weight of EKSO GT^®^ (rho = 0.335; *p* = 0.035 and rho = 0.379; *p* = 0.016), and a negative correlation between patients’ weight with the dimensions (rho = −0.384; *p* = 0.015) and durability (rho = −0.343; *p* = 0.030); and between patients’ height and dimensions (rho = −0.314; *p* = 0.049), durability (rho = −0.334; *p* = 0.035) and security (rho = −0.314; *p* = 0.048) of EKSO GT^®^ (Table 4).

The correlation analysis for physiotherapists’ questionnaire showed a moderate association between their age and the satisfaction with the device adjustment (rho = 0.685; *p* = 0.007), and an excellent association between the time of experience in neurology rehabilitation and the satisfaction with combining the device with other gait trainings (rho = 0.723; *p* = 0.003). Table 3 summarizes the correlations of the physiotherapists, and Table 4 of the patients.

Only one patient complained of low back pain days after a few sessions but not during sessions, the gait training with EKSO GT^®^ was stopped for safety. No other adverse effects were reported.

## 4. Discussion

In the present study, we evaluate the satisfaction of people with MS and their physiotherapists with the wearable exoskeleton EKSO GT^®^. In addition, we investigated the potential correlations between satisfaction and clinical data. While we are aware that some studies have evaluated satisfaction with EKSO GT^®^ in patients with spinal cord injury [35], to our best knowledge, this is the first study providing an evaluation of the satisfaction of training with EKSO GT^®^ in people with MS and the clinical staff involved.

Forty patients completed the study and the overall ratings on the satisfaction scales were high. The mean score obtained for the QUEST 2.0 scale was 31.3 ± 5.70 out of 40, which is a good general satisfaction, higher than other similar studies that also investigated satisfaction evaluated by QUEST 2.0 with other exoskeletons. Puyuelo-Quintana et al. [36] obtained a mean score of 22.4 ± 3.2 with the Marsi Active Knee^®^, a robotic knee orthosis exoskeleton developed for patients with stroke and MS. Kozlowski et al. [37] obtained a 29.36 ± 2.48 mean score with ReWalk Rehabilitation 2.0^®^ exoskeleton in people with MS. This difference might be due to the fact that in the study of Kozlowski et al. [37] the participants who attended sessions on consecutive days often performed less well in their next session, which may have decreased their satisfaction, probably due to fatigue. In our study no patient had consecutive sessions. Nevertheless, in the study of Awad et al. [38] the exosuit ReWalk ReStore™ was used in stroke patients and showed a higher mean score of 33.8 ± 8.1 on QUEST 2.0 than in the present study. The light weight of the exosuit (5 kg) compared to the high weight of the EKSO GT^®^ (27 kg) and the higher stiffness of its structure could be the reasons of the higher satisfaction from the exosuit, since the items comfort and weight are two of the items with lowest scores in our study. Moreover, the most relevant characteristic was the effectiveness in both studies. However, the second most selected characteristic differs, being comfort in the study of Awad et al. [38], and safety in our study. It should be noted that the ReWalk ReStore^®^ only supports the ankle movements, with no control of the knee and hip movements as EKSO GT^®^ offers. The item with the highest score in our study was safety, which corresponds to the almost zero presence of adverse effects. Finally, Gomez-Vargas et al. [39] studied kinematic, spatio-temporal and user satisfaction changes of the T-Flex robot in 10 ischemic stroke patients. They assessed user’s satisfaction also with QUEST questionnaire and their results showed that the level of satisfaction was between satisfied and very satisfied in 60% and 40%, respectively. The most selected parameter was comfort. However, no numerical data are showed in their paper to explore other comparisons with our findings. 

Other commercially available exoskeletons than the EKSO GT^®^ have been described in the literature such as: HAL, REX, INDEGO, Keoogo, Atalante, BLEEX, ExoAtlet I, Exo-H2, Hank, H-MEX, Mina, Phoenix, ROKI, Tréxo Plus or Wearable Power Assist Locomotor [10]. However, as it has been indicated, no satisfaction studies have been conducted with them in people with MS. Therefore, our research could be considered as a benchmark about satisfaction studies with exoskeletons in people with MS.

Satisfaction studies using other scales than QUEST 2.0 with other exoskeletons and other neurological disorders have been carried out. Platz et al. [40] studied user satisfaction in people with spinal cord injury with ReWalk. The protocol was proposed for 4-5 weeks with a daily training of 60 min. A Likert satisfaction scale was used with higher scores in comfortable, not cause considerable pain, no breathing difficulties and safety items. Finally, Bortole et al. [41] used the H2 exoskeleton and examined its safety and usability in a clinical setting under an intensive 4-week training with 3 patients with hemiparesis after a stroke. User satisfaction was assessed by a Likert scale with a mean score of 7.2 points out 10 points. Several examples of positive comments were: “the device is light”, “it is quick and simple to wear”, “it helps me with my knee flexion”, “this training is more exciting than manual gait training” or “I would have liked to have access to this device while I was in the hospital”.

Regarding the results of the physiotherapists’ satisfaction questionnaire, the mean score was 38.50 ± 3.67 points out of 45 points, proving a good degree of satisfaction. According to Read et al., [42] physiotherapists are getting used to work in technological environments, so introducing this type of technology may not present many difficulties. Question 5 showed the highest score of the questionnaire with 4.86 ± 0.35 out of 5 points, which means that physiotherapists found that the EKSO GT^®^ had a positive impact in the patients’ gait, in accordance with other studies that showed improvements in gait, balance or better activity of the trunk muscles with EKSO GT^®^ [8,43,44]. In agreement with much of the literature on robotic exoskeletons, the type of the device used could influence walking capacity [45], since it has been observed that EKSO GT^®^ allows more work during session than grounded exoskeletons such as Lokomat [35].

Our results show a moderate correlation between the number of sessions and the satisfaction score of the CSQ8 and the effectiveness item of the QUEST 2.0. Kozlowski et al. [46] found that learning needs depended on patients’ characteristics, and Puyuelo-Quintana et al. [36] reported a low satisfaction with the effectiveness because the training period was too short. Thereby, some patients would need more sessions to a properly use of the EKSO GT^®^, increasing their satisfaction with effectiveness as they perform more sessions. In addition, a moderate correlation between MSIS and the comfort and weight of EKSO GT^®^ was observed as well as a negative correlation between patients’ weight and height with the dimensions, durability, and security of EKSO GT^®^. These results may indicate that the stiff structures of EKSO GT^®^ become more uncomfortable and unsafe when the patients are at the limit of the height and weight specifications of the EKSO GT^®^ or have a greater impact of the disease.

Attending to the physiotherapists’ questionnaire, question 4 (compatibility with other gait trainings) demonstrated an excellent correlation with the years of experience in the neurorehabilitation field, and lower correlations with physiotherapists’ age and the years of experience in the rehabilitation of people with MS. Moderate correlations were observed between question 5 and physiotherapists’ age and years of experience in the neurorehabilitation field and with MS. The ability to adjust the exoskeleton to the patients’ needs such as the amount of assistance provided to optimize patients’ gait pattern is an essential skill for the physiotherapists [47]. The high score and the obtained correlation seem to indicate that EKSO GT^®^ could properly adapt to patients’ and physiotherapists’ needs.

Our results present clinical implications. A good degree of satisfaction for people with MS and physiotherapists with the EKSO GT^®^ was obtained. The parameter selected the most by the MS patients was the safety followed by effectiveness in the QUEST. This shows that easy to use and comfort items might be improved for future versions of EKSO GT^®^ for people with MS. Additionally, our results show a moderate correlation between the number of sessions and the satisfaction (CSQ8 and the effectiveness item of the QUEST 2.0.) perceived by patients, so the training period is an important element in gait rehabilitation with robotics in people with MS. Attending to the physiotherapists’ questionnaire, the compatibility with other gait and the years of experience in the neurorehabilitation field could be an important combination to achieve higher satisfaction for this collective. Finally, as patient motivation can be modified by several processes, such as increasing problem awareness and information in patients, involving them in the design and implementation of the treatment program, enhancing their level of internal control and raising their hope of recovery [48], future designs should consider our findings about exoskeleton robots in people with MS. Other tasks, such as stairs, turning and bending should be explored in future designs as they are very related to functional tasks.

Our study presents several limitations. Firstly, we must report the difference in the number of sessions per week performed by each patient. This difference in the training intensity may affect the effectiveness of the treatment, resulting indifferent satisfaction perceptions. Future research should control the intensity of sessions to increase the soundness of the findings. Second, our results cannot be overextended for all patients with MS, therefore it is necessary to understand these findings with caution and to take the EDSS scores into consideration. Moreover, future studies could be conducted to reduce the selection bias as all patients were recruited from the same clinical setting. Finally, future studies are needed incorporating a control group. It should be noticed that QUEST questionnaire is designed to measure satisfaction with technological devices. If a control group is recruited under conventional rehabilitation treatment, other general questionnaires are needed, as a comparison with different approaches would be conducted. 

## 5. Conclusions

This study demonstrates a good degree of satisfaction for people with MS and physiotherapists with the EKSO GT^®^. Effectiveness and safety were the most highly rated characteristics of the EKSO GT^®^ according to the patients. The most valued characteristic of the EKSO GT^®^ for the physiotherapists involved was its positive impact on the patients’ gait and the compatibility with other gait trainings.

A correlation between the number of sessions and the patients’ satisfaction was obtained. Furthermore, age and years of experience in the neurorehabilitation field were correlated with physiotherapists’ satisfaction. Features such as comfort or weight of the device should be improved from the patients’ perspectives, with greater height and weight applicability in future modifications of the technical design.

## Figures and Tables

**Table 1:** Physiotherapists’ satisfaction questionnaire.

How Much Do You Agree or Disagree with the Following Statements?	
1. The amount of time spent adjusting to the device is short for clinical practice.	1 2 3 4 5
2. Device settings can be correctly adjusted to the needs of each patient.	1 2 3 4 5
3. The use of the device does not interfere with the supervision and protection of the patient during sessions.	1 2 3 4 5
4. The device is compatible with other gait trainings.	1 2 3 4 5
5. The device has a positive impact in the patients’ gait.	1 2 3 4 5
6. The device is useful in clinical practice.	1 2 3 4 5
7. The information provided by the device is easy to understand.	1 2 3 4 5
8. The assistance provided by the device to correct the patients is clear (visual and sound feedback).	1 2 3 4 5
9. I would recommend this device to other physiotherapists.	1 2 3 4 5

**Table 2:** Mean score of the different scales.

Measurement	Mean ± SD	Measurement	Mean ± SD
MSWS	46.33 ± 9.87	QUEST 2.0 Dimensions	4.08 ± 0.93
MSIS Physical	64.3 ± 18.24	QUEST 2.0 Weight	3.80 ± 0.95
MSIS Psychological	21.5 ± 7.38	QUEST 2.0 Adjustments	3.80 ± 1.00
MSIS Total	85.8 ± 23.28	QUEST 2.0 Safety	4.25 ± 0.89
MFIS Physical	24.78 ± 7.51	QUEST 2.0 Durability	3.98 ± 0.96
MFIS Cognitive	13.28 ± 9.56	QUEST 2.0 Easy to Use	3.73 ± 0.92
MFIS Social	4.33 ± 2.18	QUEST 2.0 Comfort	3.65 ± 1.04
MFIS Total	42.38 ± 16.18	QUEST 2.0 Effectiveness	4.03 ± 1.01
SF36 Physical Functioning	21.5 ± 20.61	QUEST 2.0 Total (40)	31.3 ± 5.70
SF36 Physical Role	42.34 ± 29.23	CSQ8	26.28 ± 4.68
SF36 Bodily Pain	57.5 ± 27.15		
SF36 General Health	35.75 ± 20.46		
SF36 Social Functioning	63.44 ± 26.91		
SF36 Vitality	37.5 ± 21.93		
SF36 Emotional Role	82.5 ± 25.02		
SF36 Mental Health	73.63 ± 17.10		
SF36 Total	48.91 ± 15.48		
HADS Anxiety	5.2 ± 3.32		
HADS Depression	5.18 ± 3.23		

QUEST: Quebec User Evaluation with Assistive Technology; CSQ8: Client Satisfaction Questionnaire; MSWS: Multiple Sclerosis Walking Scale; MSIS: Multiple Sclerosis Impact Scale; MFIS: Modified Fatigue Impact Scale; SF36: Short Form Health Survey; HADS: Hospital Anxiety and Depression Scale; SD: standard deviation.

**Table 3:** Physiotherapists’ satisfaction scale score and correlation with physiotherapists’ data.

Questions	Mean ± SD	Gender			Age			Years NR			Years MS		
		rho	CI	*p*	rho	CI	*p*	rho	CI	*p*	rho	CI	*p*
Question 1	3.71 ± 0.88	0.394	−0.173 to 0.765	0.163	0.151	−0.413 to 0.631	0.607	0.372	−0.198 to 0.754	0.190	0.227	−0.345 to 0.676	0.434
Question 2	4.29 ± 0.59	0.144	−0.418 to 0.627	0.623	0.685 **	**0.243 to 0.892**	**0.007**	0.622 *	**0.136 to 0.867**	**0.018**	0.655 *	**0.191 to 0.880**	**0.011**
Question 3	3.93 ± 0.96	0.477	−0.071 to 0.804	0.084	0.224	−0.348 to 0.674	0.442	0.317	−0.257 to 0.725	0.270	0.133	−0.428 to 0.620	0.651
Question 4	4.71 ± 0.59	0.212	−0.359 to 0.667	0.467	0.593 *	**0.091 to 0.855**	**0.026**	0.723 **	**0.312 to 0.906**	**0.003**	0.580 *	**0.071 to 0.849**	**0.030**
Question 5	4.86 ± 0.35	0.167	−0.399 to 0.641	0.569	**0.564** *	**0.048 to 0.842**	**0.036**	0.510	−0.028 to 0.819	0.062	0.358	−0.213 to 0.747	0.208
Question 6	4.43 ± 0.62	0.396	−0.170 to 0.766	0.161	0.175	−0.392 to 0.646	0.549	−0.021	−0.546 to 0.515	0.943	0.298	−0.276 to 0.716	0.300
Question 7	4.00 ± 0.53	0.382	−0.186 to 0.759	0.178	−0.235	−0.681 to 0.338	0.419	0.084	−0.468 to0.588	0.777	−0.050	−0.566 to 0.493	0.864
Question 8	4.00 ± 0.53	0.382	−0.186 to 0.759	0.178	0.252	−0.322 to 0.690	0.385	0.384	−0.184 to 0.760	0.175	−0.084	−0.588 to 0.468	0.776
Question 9	4.57 ± 0.62	0.300	−0.275 to 0.716	0.298	0.517	−0.018 to 0.822	0.058	0.525	−0.007 to 0.826	0.054	0.456	−0.098 to 0.794	0.101

CI: confidence interval; Years NR: time of experience in neurology rehabilitation; Years MS: time of experience in rehabilitation in people with Multiple Sclerosis. * = *p* < 0.05 (bold values) using Rho of Spearman. ** = *p* < 0.01 (bold values) using Rho of Spearman.

**Table 4:** Correlation between satisfaction scales and data from patients.

	Age			Height			Weight			YearsD			EDSS			Sessions		
QUEST 2.0	rho	CI	*p*	rho	CI	*p*	rho	CI	*p*	rho	CI	*p*	rho	CI	*p*	rho	CI	*p*
Dimensions	0.118	−0.201 to 0.414	0.468	−0.314 *	**−0.570 to −0.003**	**0.049**	−0.384 *	**−0.621 to −0.082**	**0.015**	0.151	−0.168 to 0.442	0.352	0.024	−0.290 to 0.333	0.882	0.107	−0.212 to 0.405	0.511
Weight	0.016	−0.297 to 0.326	0.920	−0.036	−0.344 to 0.279	0.827	0.064	−0.253 to 0.368	0.694	0.013	−0.300 to 0.323	0.937	0.216	−0.102 to 0.494	0.181	0.201	−0.118 to 0.482	0.213
Adjustments	0.082	−0.236 to 0.384	0.616	−0.215	−0.493 to 0.103	0.183	−0.149	−0.440 to 0.170	0.359	−0.056	−0.361 to 0.260	0.732	0.062	−0.254 to 0.366	0.705	0.174	−0.145 to 0.461	0.284
Safety	−0.070	−0.373 to 0.247	0.668	−0.314 *	**−0.570 to −0.003**	**0.048**	−0.281	−0.545 to 0.033	0.078	−0.076	−0.379 to 0.241	0.639	0.050	−0.266 to 0.356	0.761	0.257	−0.059 to 0.526	0.110
Durability	0.111	−0.208 to 0.408	0.497	−0.334 *	**−0.585 to −0.025**	**0.035**	−0.343 *	**−0.591 to −0.035**	**0.030**	−0.055	−0.360 to 0.261	0.734	−0.070	−0.373 to 0.247	0.666	0.173	−0.146 to 0.460	0.286
Easy to Use	−0.165	−0.453 to 0.154	0.309	0.073	−0.244 to 0.376	0.655	−0.005	−0.316 to 0.307	0.976	−0.028	−0.337 to 0.286	0.866	−0.053	−0.359 to 0.263	0.745	0.208	−0.111 to 0.488	0.199
Comfort	−0.149	−0.440 to 0.170	0.357	0.087	−0.231 to 0.388	0.592	−0.148	−0.439 to 0.171	0.362	0.031	−0.283 to 0.339	0.848	0.139	−0.180 to 0.432	0.393	0.198	−0.121 to 0.480	0.220
Effectiveness	−0.061	−0.366 to 0.255	0.709	−0.239	−0.512 to 0.078	0.137	−0.255	−0.525 to 0.061	0.113	−0.092	−0.392 to 0.226	0.573	−0.071	−0.374 to 0.246	0.664	0.357 *	**0.051 to 0.602**	**0.024**
Total	−0.030	−0.338 to 0.284	0.856	−0.233	−0.508 to 0.085	0.147	−0.267	−0.534 to 0.049	0.095	−0.033	−0.341 to 0.281	0.841	0.027	−0.287 to 0.336	0.869	0.265	−0.051to0.533	0.098
**CSQ8**	0.053	−0.263 to 0.359	0.747	−0.157	−0.447 to 0.162	0.333	−0.104	−0.402 to 0.214	0.524	−0.053	−0.359 to 0.263	0.747	−0.107	−0.405 to 0.212	0.511	0.532 **	**0.264 to 0.724**	**<0.001**
	**MSWS**			**MSIS**			**MFIS**			**SF36**			**HADS Anxiety**			**HADS Depression**		
**QUEST 2.0**	**rho**	**CI**	***p***	**rho**	**CI**	***p***	**rho**	**CI**	***p***	**rho**	**CI**	***p***	**rho**	**CI**	***p***	**rho**	**CI**	***p***
Dimensions	0.144	−0.228 to 0.479	0.448	0.066	−0.251 to 0.370	0.686	−0.145	−0.437 to 0.174	0.371	−0.004	−0.315 to 0.308	0.980	0.148	−0.171 to 0.439	0.361	0.090	−0.228 to 0.391	0.583
Weight	0.322	−0.043 to 0.611	0.083	0.335 *	**0.026 to 0.585**	**0.035**	0.270	−0.045 to 0.536	0.092	−0.161	−0.450 to 0.158	0.322	0.169	−0.150 to 0.456	0.297	0.065	−0.252 to 0.369	0.692
Adjustments	0.194	−0.179 to 0.518	0.305	0.174	−0.145 to 0.461	0.282	0.103	−0.215 to 0.402	0.526	−0.077	−0.379 to 0.240	0.637	0.204	−0.115 to 0.485	0.208	0.086	−0.232 to 0.387	0.600
Safety	−0.109	−0.452 to 0.262	0.567	0.060	−0.256 to 0.365	0.713	−0.186	−0.470 to 0.133	0.249	0.178	−0.141 to 0.464	0.272	−0.065	−0.369 to 0.252	0.692	0.001	−0.311 to 0.312	0.993
Durability	−0.005	−0.365 to 0.356	0.979	0.152	−0.167 to 0.443	0.350	−0.007	−0.318 to 0.305	0.965	−0.086	−0.387 to 0.232	0.596	0.142	−0.177 to 0.434	0.381	0.184	−0.135 to 0.469	0.256
Easy to Use	−0.252	−0.561 to 0.119	0.180	0.085	−0.233 to 0.386	0.601	0.096	−0.222 to 0.396	0.554	−0.078	−0.380 to 0.239	0.633	0.153	−0.166 to 0.443	0.345	0.135	−0.184 to 0.428	0.407
Comfort	−0.194	−0.518 to 0.179	0.305	0.379 *	**0.077 to 0.618**	**0.016**	0.274	−0.041 to 0.539	0.088	−0.181	−0.466 to 0.138	0.263	0.304	−0.008 to 0.562	0.057	0.096	−0.222 to 0.396	0.557
Effectiveness	−0.171	−0.500 to 0.202	0.365	−0.186	−0.470 to 0.133	0.251	−0.335 *	**−0.585 to −0.026**	**0.035**	0.289	−0.025 to 0.551	0.071	−0.194	−0.477 to 0.125	0.231	0.023	−0.291 to 0.332	0.886
Total	−0.018	−0.376 to 0.345	0.923	0.147	−0.172 to 0.438	0.365	0.029	−0.285 to 0.337	0.859	−0.014	−0.324 to 0.299	0.932	0.173	−0.146 to 0.460	0.287	0.105	−0.213 to 0.403	0.517
**CSQ8**	0.060	−0.307 to 0.411	0.752	−0.087	−0.388 to 0.231	0.593	−0.202	−0.483 to 0.117	0.212	0.102	−0.216 to 0.401	0.532	−0.059	−0.364 to 0.257	0.718	−0.002	−0.313 to 0.310	0.989

CI: confidence interval; Dim: dimensions; QUEST: Quebec User Evaluation with Assistive Technology; CSQ8: Client Satisfaction Questionnaire; YearsD: years since diagnosis; SWS: Multiple Sclerosis Walking Scale; MSIS: Multiple Sclerosis Impact Scale; MFIS: Modified Fatigue Impact Scale; SF36: Short Form Health Survey; HADS: Hospital Anxiety and Depression Scale. * = *p* < 0.05 (bold values) using Rho of Spearman. ** = *p* < 0.01 (bold values) using Rho of Spearman.
